# Ensuring equitable emergency, critical and operative care

**DOI:** 10.1186/s41043-026-01472-x

**Published:** 2026-10-20

**Authors:** Tim Baker, Carl Otto Schell, Pauline Convocar, Fitsum Kifle, Niranjan Kissoon, Nobhojit Roy, Karima Khalid

**Affiliations:** 1 Department of Global Public Health, Karolinska Institutet, Stockholm, Sweden; 2 Muhimbili University of Health and Allied Sciences, Dar es Salaam, Tanzania; 3 International Federation of Emergency Medicine, Manila, Philippines; 4 Debre Berhan University, Addis Ababa, Ethiopia; 5 Department of Pediatrics, University of British Columbia, Vancouver, Canada; 6 George Institute for Global Health, Mumbai, India

## Abstract

Conditions requiring emergency, critical and operative (ECO) care account for a substantial proportion of preventable mortality and disability worldwide, yet ECO systems remain fragmented and underdeveloped in many settings. Recent global policy advances, including the adoption of the World Health Organization (WHO) Global Strategy and Action Plan for Integrated ECO Care by the World Health Assembly, provide an important opportunity to strengthen these systems. However, implementation must prioritise equity. ECO care spans a wide spectrum of complexity and cost, from basic triage, oxygen therapy and essential operations to advanced intensive care and specialised surgery. Given finite resources, health systems should first ensure universal access to foundational ECO care: early recognition of illness, basic airway and oxygen support, essential medications, monitoring, infection prevention, and timely referral. Approaches such as Essential Emergency and Critical Care (EECC) offer a feasible, highly cost-effective minimum standard of care, and prioritising such foundational ECO care is both an ethical imperative and the most effective pathway to achieving equitable population-level health gains.

## Introduction

Conditions requiring emergency, critical and operative (ECO) care are among the largest causes of preventable death and disability worldwide. Emergency conditions account for 51% of deaths and 42% of disability-adjusted life years (DALYs) globally [1]. There are more than 45 million cases of critical illness each year [2] and one in eight hospitalised patients is critically ill [3, 4]. Five billion people do not have access to safe, affordable surgical and anaesthesia care [5], and a similar number do not have access to safe, affordable oxygen [6]. Despite this, systems for ECO care have historically received limited attention and remain fragmented and underdeveloped in many settings.

Cognizant of this oversight, at the World Health Assembly resolution 76.2 in 2023 calling for a strengthening of health systems for high-quality ECO care passed with unanimous support. In 2024, decision 77[8] [ tasked the WHO with developing a global strategy and action plan for integrated ECO care, and in May this year the global strategy and action plan were adopted by the 79th World Health Assembly. These are vital milestones that provide hope and momentum that ECO care will be strengthened and improved globally.

ECO care can be provided at vastly different levels of complexity and cost, from foundational triage at arrival to a health facility, basic resuscitation, oxygen therapy and essential operations to advanced multi-organ resuscitation, intensive care, and super-specialised surgery. ECO care can cost as little as $17USD to as much as thousands of dollars per day [7]. Health systems vary and context matters such that the level and intensity of care offered must be decided locally. However, to maximise equity and efficiency, we must prioritise among finite resources and ensure that all systems provide at least the most foundational care – the care that all ECO patients need. This is the principle in WHO’s Fair Choices to prioritise equity on the path to Universal Health Coverage [8]. A potential risk to ECO system development is overinvestment in advanced services that will only benefit a few, while foundational care for all remains underdeveloped. If implementation prioritises the foundational care, ECO system strengthening can be both equitable and impactful.

### Essential Emergency and Critical C are

EECC is cheap and can be highly cost-effective. At $14USD per DALY averted it can have similar cost-effectiveness to anti-malarial drugs and vaccinations [11]. The WHO and member states should be commended on the much-needed push to improve ECO systems. We call on policy makers, health system managers, and global health stakeholders to ensure that every person, everywhere, receives at least the most essential emergency, critical, and operative care. Making the foundational ECO care the priority is achievable, is a moral imperative, and is the surest route to equitable, population-level impact with health systems that serve us all.

**Fig. 1:**
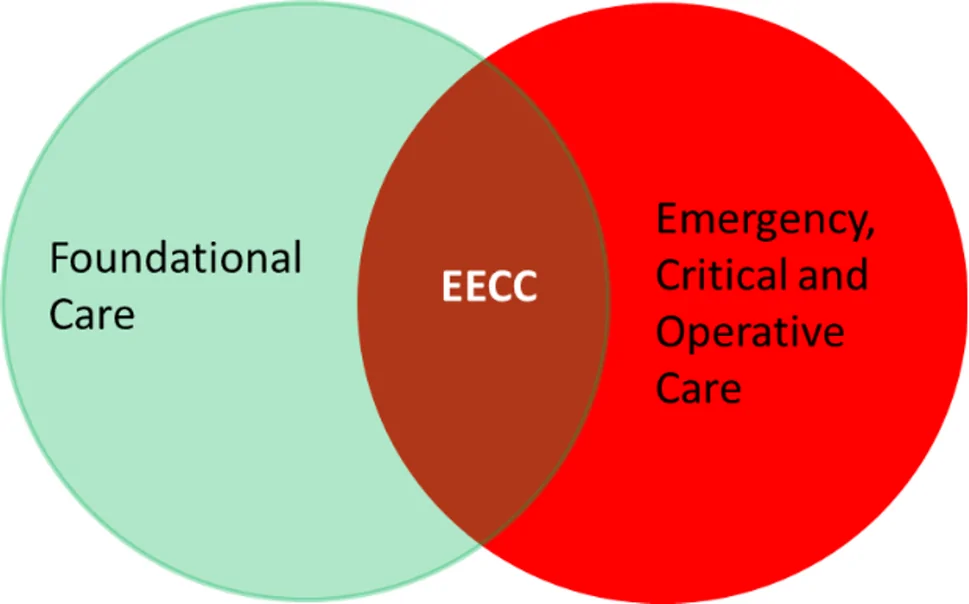
Foundational ECO Care. EECC: Essential Emergency and Critical Care

Foundational ECO care includes early recognition of illness, basic airway support, oxygen therapy, essential medications, monitoring of vital signs, infection prevention, and appropriate escalation or referral (Fig. 1). Several ongoing global and national initiatives are working towards their implementation. The WHO/ICRC Basic Emergency Care course is being rolled out in multiple countries, and a complementary Basic Critical Care course is in development. At the system level, Essential Emergency and Critical Care (EECC) is a minimum standard: the clinical processes and resources needed for the most foundational care for all critically ill patients including in the perioperative period [9]. Several nations have integrated EECC into their health systems and the World Federation of Societies of Anaesthesia has called for countries to ensure that all critically ill patients receive EECC [10]. 

## Data Availability

No datasets were generated or analysed during the current study.

## Abbreviations

DALY: Disability adjusted life-year
ECO: Emergency, critical and operative
EECC: Essential emergency and critical care
ICRC: International committee of the red cross
WHO: World health organisation
